# Nonprehensile Manipulation for Rapid Object Spinning via Multisensory Learning from Demonstration

**DOI:** 10.3390/s24020380

**Published:** 2024-01-08

**Authors:** Ku Jin Shin, Soo Jeon

**Affiliations:** 1 CrowdRiff, 225 King St W Suite 1200, Toronto, ON M5V 3M2, Canada; kj2shin@uwaterloo.ca; 2 Department of Mechanical & Mechatronics Engineering, University of Waterloo, 200 University Ave. W., Waterloo, ON N2L 3G1, Canada

**Keywords:** multimodal sensing, dexterous manipulation, learning from demonstration

## Abstract

Dexterous manipulation concerns the control of a robot hand to manipulate an object in a desired manner. While classical dexterous manipulation strategies are based on stable grasping (or force closure), many human-like manipulation tasks do not maintain grasp stability and often utilize the dynamics of the object rather than the closed form of kinematic relation between the object and the robotic hand. Such manipulation strategies are referred as nonprehensile or dynamic dexterous manipulation in the literature. Nonprehensile manipulation often involves fast and agile movements such as throwing and flipping. Due to the complexity of such motions and uncertainties associated with them, it has been challenging to realize nonprehensile manipulation tasks in a reliable way. In this paper, we propose a new control strategy to realize practical nonprehensile manipulation. First, we make explicit use of multiple modalities of sensory data for the design of control law. Specifically, force data are employed for feedforward control, while position data are used for feedback control. Secondly, control signals (both feedback and feedforward) are obtained through multisensory learning from demonstration (LfD) experiments designed and performed for specific nonprehensile manipulation tasks of concern. To prove the concept of the proposed control strategy, experimental tests were conducted for a dynamic spinning task using a sensory-rich, two-finger robotic hand. The control performance (i.e., the speed and accuracy of the spinning task) was also compared with that of classical dexterous manipulation based on force closure and finger gaiting.

## 1. Introduction

Human-like dexterous manipulation of robotic hands has long been recognized as a critical challenge to the next generation of robots [1,2]. Much effort has been made to realize some hand manipulations, such as regrasping, in-grasp manipulation, finger gating, finger pivoting/tracking, rolling, and sliding [3,4,5]. Historically, grasp stability has been considered a basic requirement of dexterous manipulation with multifingered robotic hands [6].

Instead of relying on grasp stability, an alternative approach is to manipulate objects without maintaining a stable grasp; such manipulation is referred as nonprehensile (or dynamic dexterous) manipulation [7]. Nonprehensile manipulation offers several potential benefits over conventional approaches, such as increased dexterity and increased workspace [8]. This enables robots to perform more human-like manipulations, such as pushing, throwing, and flipping [9]. The key aspect of nonprehensile manipulation is the utilization of the intrinsic dynamics of the target object (even while the object is not in contact with the manipulating bodies).

There have been some notable attempts to realize nonprehensile manipulation with robotic hands [10,11], most of which have relied on mathematical modeling of the hand and the object, as well as the dynamics between them. Despite some successes, the complexity of dynamic models and uncertainties associated with them have been a major technical hurdle to taking dexterous manipulation to the next level. Control design can also become inefficient with such model-based approaches due to difficulty in handling variabilities arising from a changing environment (e.g., variability in objects and/or task goals). The problem we address in this paper is how we can overcome these limitations faced by conventional model-based approaches by incorporating the demonstration data from a human expert and, thus, how we can achieve more agile and fast nonprehensile manipulation. Furthermore, we aim to leverage different modalities of sensory data available from various sensors including visual and tactile sensation to make full use of modern robotic sensing technologies.

More specifically, we incorporate two main ideas in this paper. First, we employ recent techniques of learning from demonstration (LfD) for nonprehensile manipulation. To the best of our knowledge, LfD has not yet been implemented for the type of nonprehensile manipulation tasks that we consider in this paper. Secondly, in doing so, we make the explicit use of multiple modalities of sensory data in formulating the control law for LfD, i.e., visual images and fingertip force data. In fact, these two ideas parallel our own intuition (or hypotheses) of how human dexterity develops [12,13] through visuotactile integration.

As an active area of research in robotics, LfD has been the subject of significant progress in recent years [14,15]. LfD has also been referred to as imitation learning, programming by demonstration (PbD), and apprenticeship learning (AL) in the literature [16]. LfD is an autonomous learning strategy through which a non-expert human can configure a robot to perform a complex task by providing a good set of demonstrations of the desired task. This technique has been shown to hold a great potential for the control of a robot hand without direct programming or configuration.

Thanks to rapid advances in sensing technologies, modern robotic systems are increasingly equipped with various sensors, such as motor encoders, strain gauges, tactile sensors, high-speed vision cameras, or a combination thereof. The concept of sensory-rich control (or motion control using multimodal sensory data) has been implemented in some classical control applications [17,18], but its practical use for dexterous manipulation has not yet been fully exploited.

In this work, we examine the performance of nonprehensile manipulation based on the LfD approach using a custom-built robotic hand. The robotic hand is equipped with various sensors, including tactile (or fingertip force) and vision sensors, as well as position encoders. As an exemplary task, we realized a nonprehensile task of spinning and stopping a rigid circular disk with two fingers, the goal of which is to make the disk rotate as fast and as close to the desired angle as possible. Using the fingertip force sensors and position encoders, the expert’s demonstration data are collected. Using Gaussian mixture regression (GMR), the position and fingertip force data were processed to find the Gaussian mixture models (GMMs) for the desired position and the desired contact force signals, respectively. The desired position signal is used for feedback control, while the desired contact force signal is injected as a feedforward command. The performance of the proposed control strategy was verified in comparison with that of the traditional regrasping method in the spinning task.

Based on our discussions presented above, we can summarize the main contributions of this paper as follows:

(1) In order to realize more agile and fast nonprehensile manipulation, in this paper, we propose a systematic way to generate reliable and efficient reference trajectories through LfD. This is in contrast to conventional approaches where desired motion profiles are obtained from mathematical models of the manipulator and the target object.
(2) We make explicit use of multimodal sensory data (i.e., vision, force, and position data) for both LfD (i.e., reference trajectory generation) and motion control processes. Compared to approaches based only on positional signal, our strategy can be particularly useful for nonprehensile manipulation involving impulsive actions such as the one considered in this paper.

## 2. Related Works

In-hand manipulation has long been studied in the literature. Previous works include that by Salisbury [19]. A good classic review on the topic can be found in [1,20]. Despite such a long history, the problems of the manipulating position and orientation of objects with human-like dexterity remain a considerable challenge [2,21,22]. The representative types of dexterous manipulation tasks that have been achieved so far using an anthropomorphic robot hand include regrasping/finger gaiting [23] and sliding/rolling [24]. Regrasping/finger gaiting involves the sequence of steps of grasping and releasing an object. The main drawback of this type of manipulation is that an additional support plane is required to hold the object when it is detached from the robot. Also, it takes more time to manipulate the object due to the large number of sequences of actions required to achieve the desired task. Sliding/rolling has been pursued as an alternative strategy to perform in-hand manipulation. It may enable faster and more agile manipulations in some cases by allowing the object to slide or roll against the fingers (often without grasp stability) during the manipulation [25]. Many in-hand manipulation approaches pursue a model-based planning perspective, i.e., grasp planning and motion planning based on kinematic relations. A majority of methods presume that the analytical descriptions of both the hand and the object are known and that the force-closure condition is maintained [6]. In this paper, we are interested in developing a general control strategy that can achieve highly dexterous manipulation tasks without precise description of the models of the hand and the object.

Control laws for dexterous manipulation have largely relied on precise knowledge of the position of the manipulating fingers and/or the target object. Other types of sensory data adopted in recent years include tactile and vision data [26,27]. Closing a feedback loop with such sensors may enable the control law to adapt to unknown object properties [28]. In particular, tactile sensory information can play an important role in the process of manipulation by detecting both the direction and the magnitude of the contact force between the object and the robotic fingers. Examples of the utility of tactile information in in-hand manipulation tasks can be found in [29,30,31]. As is the case for humans, the visual sense can serve another important sensory feedback purpose in dexterous manipulation. Visual servoing—or vision-based robot control—is well-established in the literature and can enable precise manipulation with both fully actuated hands [32,33] and underactuated hands [34].

The incorporation of demonstration data from human experts into robot task control has been extensively investigated in recent years. There exist many different approaches, but one view is that they fall into one of two main categories: reinforcement learning (RL) methods and LfD methods. RL requires predefined reward or cost function information. Coming up with a well-defined function is crucial, since this governs the performance of the learning. LfD is a more common approach, allowing robots to perform human tasks based on demonstration data. Many proposals adopt a probabilistic approach to encode task variables using a probability density function (pdf) and reproduce them using regression techniques. The combination of a Gaussian mixture model and the Gaussian mixture regression (GMM/GMR) technique is one of the most widely used probabilistic approaches in the field [35]. There also exist some extensions of GMM/GMR, such as the task-parameterized version of GMM (TP-GMM) [36]. Most of the problems associated with LfD involve learning the position trajectory of the robot to perform the desired task; however, some researchers have expanded the idea to force-based manipulation tasks [37,38,39,40]. In this paper, we attempt to combine the advantages of position-based and force-based GMM/GMR.

## 3. Background

In this section, we briefly review some background theories of the LfD framework that we employ in this paper.

### 3.1. Gaussian Mixture Model

A Gaussian mixture model (GMM) is a probabilistic model that assumes that all of the data points are generated from a mixture of a finite number of Gaussian distributions [16]. Such a modeling technique is useful when attempting to identify a trend among multiple datasets. This can be seen as a generalized version of the k-means clustering technique [41] but may be considered more general in terms of flexibility in choosing the covariance between the Gaussian distributions. GMM can estimate the probability density distribution of the samples, where the estimated model is the weighted sum of several Gaussian models. *K* denotes the number of component Gaussian distributions, and the probability that the *D*-dimensional *j*-th data point ( $\xi_{j}\in R^{D}$ ) belongs to the GMM can be expressed as

(1) $$p\left(\xi_{j}\right)=\sum_{k=1}^{K}\pi_{k}p\left(\xi_{j}|k\right)$$

where $\pi_{k}\in \left[0,\;1\right]$ is the prior probability of the *k*-th Gaussian, and $p(\xi_{j}|k)$ is the conditional probability density function (pdf) for $\xi_{k}$ with respect to the *k*-th Gaussian under the Gaussian distribution ( $N(\xi_{j}|\mu_{k},\Sigma_{k})$ ), i.e.,

(2) $$p\left(\xi_{j}|k\right)={\frac{1}{\sqrt{{\left(2\pi \right)}^{D}\left|\Sigma_{k}\right|}}}^{-\frac{1}{2}\left({\left(\xi_{j}-\mu_{k}\right)}^{⊺}\left(\theta \right)\Sigma_{k}^{-1}\left(\xi_{j}-\mu_{k}\right)\right)}$$

where $\mu_{k}$ and $\Sigma_{k}$ denote the mean and the covariance matrix for the *k*-th Gaussian, respectively. Thus, the GMM can be characterized by the following set of parameters: $\Theta^{\mathrm{GMM}}={\left\{\pi_{k},\mu_{k},\Sigma_{k}\right\}}_{k=1}^{K}$ . The prior probability ( $\pi_{k}$ ) acts as a weighting factor for each Gaussian model, and it satisfies $\sum_{k=1}^{K}\pi_{k}=1$ . These sets of unknown parameters can be found using the standard expectation maximization (EM) algorithm, which is basically the iteratively performed maximum likelihood estimation (MLE) of the mixture parameters [42]. To this end, let us first define a set of posterior probabilities (called responsibilities) for a given datapoint value ( ξ ) using Baye’s rule:

(3) $$\gamma_{k}\left(\xi \right)=p\left(k|\xi \right)=\frac{p(k)p(\xi |k)}{p(\xi )}=\frac{\pi_{k}N\left(\xi |\mu_{k},\Sigma_{k}\right)}{\sum_{k^{\prime }=1}^{K}\pi_{k}N\left(\xi |\mu_{k^{\prime }},\Sigma_{k^{\prime }}\right)}$$

As the name of the algorithm suggests, the EM algorithm iterates over two steps: the E step and the M step. During each cycle, the E step estimates the distribution of the hidden variable, given the data and the current value of the parameters; then, the M step maximizes the joint distribution of the data and the hidden variable. In other words, new data points are drawn from the given GMM, and the GMM parameters are newly calculated through the MLE step. The initial parameters are usually approximated using the *k*-means clustering algorithm [41].

E step:

$$\begin{array}{c} \forall k,j,\;\mathrm{calculate}\;\gamma_{k}\left(\xi_{j}\right)\;\mathrm{with}\;\mathrm{the}\;\mathrm{current}\;\mathrm{parameters}. \end{array}$$

M step:

$$\begin{array}{c} \begin{array}{cc} \pi_{k}^{new} & =\frac{\sum_{j=1}^{N}\gamma_{k}\left(\xi_{j}\right)}{N},\\ \mu_{k}^{new} & =\frac{\sum_{j=1}^{N}\gamma_{k}\left(\xi_{j}\right)\xi_{j}}{\sum_{j=1}^{N}\gamma_{k}\left(\xi_{j}\right)},\\ \Sigma_{k}^{new} & =\frac{\sum_{j=1}^{N}\gamma_{k}\left(\xi_{j}\right)\left(\xi_{j}-\mu_{k}^{new}\right){\left(\xi_{j}-\mu_{k}^{new}\right)}^{⊺}}{\sum_{j=1}^{N}\gamma_{k}\left(\xi_{j}\right)} \end{array} \end{array}$$

After calculating the new parameters, the log likelihood (denoted by L below) is calculated for comparison with the previous log-likelihood value such that if the increase in the log likelihood is small, then the iteration stops.

(4) $$L\left(\xi |\Theta^{\mathrm{GMM}}\right)=\sum_{n=1}^{N}\ln\left\{\sum_{k=1}^{K}w_{k}N\left(\xi_{n}|\mu_{k},\Sigma_{k}\right)\right\}$$

The stopping criterion is expressed by $\frac{L^{new}}{L}<C$ , where *C* is a threshold value.

In this paper, GMM is used to parameterize each of the demonstration datasets. For example, one set of position data (i.e., time series data) collected from demonstration by a human expert can be mathematically expressed by $p(\xi_{j})$ , where *j* indicates each sample time step, and *K* corresponds to a number of segments of the demonstration signal, each of which can be approximated by a single Gaussian pdf.

### 3.2. Gaussian Mixture Regression

From the learned GMM, we can reproduce a generalized trajectory through the Gaussian mixture regression (GMR) process [16].

Let us assume that the *j*-th data point ( $\xi_{j}$ ) consists of the input vector ( $\xi_{j}^{I}$ ) and the output vector ( $\xi_{j}^{O}$ ), i.e., $\xi_{j}=\mathrm{col}$ $\left(\xi_{j}^{I},\xi_{j}^{O}\right)$ . Accordingly, the mean vector ( $\mu_{k}$ ) and the covariance matrix ( $\Sigma_{k}$ ) in (2) can be partitioned as

(5) $$\mu_{k}=\left[\begin{array}{c} \mu_{k}^{I}\\ \mu_{k}^{O} \end{array}\right],\Sigma_{k}=\left[\begin{array}{cc} \Sigma_{k}^{I} & \Sigma_{k}^{\mathrm{IO}}\\ \Sigma_{k}^{\mathrm{OI}} & \Sigma_{k}^{O} \end{array}\right]$$

Then, the GMR process involves the prediction of the distribution of the output data ( $\xi_{j}^{O}$ ) for the *k*-th Gaussian when the input data ( $\xi^{I}$ ) are given. Specifically, using the conditional probability distribution, we have

(6) $$p\left(\xi_{j}^{O}|\xi_{j}^{I},k\right)∼N\left({\hat{\xi }}_{k,j},{\hat{\Sigma }}_{k}\right)$$

where ${\hat{\xi }}_{k,j}$ and ${\hat{\Sigma }}_{k}$ are the predicted mean covariance of $\xi_{j}^{O}$ for the *k*-th Gaussian, given the input ( $\xi_{j}^{I}$ ), respectively. Using conditional probability, they are expressed by

$$\begin{array}{cc} {\hat{\xi }}_{k,j} & =\mu_{k}^{O}+\Sigma_{k}^{\mathrm{IO}}{\left(\Sigma_{k}^{I}\right)}^{-1}\left(\xi_{j}^{I}-\mu_{k}^{I}\right),\\ {\hat{\Sigma }}_{k} & =\Sigma_{k}^{O}-\Sigma_{k}^{\mathrm{OI}}{\left(\Sigma_{k}^{I}\right)}^{-1}\Sigma_{k}^{\mathrm{IO}} \end{array}$$

Then, the complete GMM can be obtained by summing up (6) over *k* as

(7) $$p\left(\xi_{j}^{O}|\xi_{j}^{I}\right)∼\sum_{k=1}^{K}h_{k,j}N\left({\hat{\xi }}_{k,j},{\hat{\Sigma }}_{k}\right)$$

where $h_{k,j}=p\left(k|\xi_{j}^{I}\right)$ is the probability that the *k*-th Gaussian distribution is responsible for $\xi_{j}^{I}$ :

(8) $$h_{k,j}=\frac{\pi_{k}p\left(\xi_{j}^{I}|k\right)}{\sum_{i=1}^{K}\pi_{i}p\left(\xi_{j}^{I}|i\right)}$$

By using the linear transformation property of Gaussian distributions, the conditional expectation ( $p\left(\xi_{j}^{O}|\xi_{j}^{I}\right)$ ) can be approximated by a single Gaussian distribution ( $N\left({\hat{\xi }}_{j},{\hat{\Sigma }}_{j}\right)$ where ${\hat{\xi }}_{j}$ and ${\hat{\Sigma }}_{j}$ are the weighted sums of ${\hat{\xi }}_{k,j}$ and ${\hat{\Sigma }}_{k}$ , respectively) through $h_{k,j}$ over *k* [35]. In the end, the sequence of ${\hat{\xi }}_{j}$ represents the desired trajectory from the GMR, with its uncertainty (or variability) encoded by ${\hat{\Sigma }}_{j}$ for each data point (*j*).

### 3.3. Dynamic Time Warping

The GMM/GMR approach explained above is capable of capturing spatial variability. However, it does not effectively encapsulate the temporal variation within the dataset. The dynamic time warping (DTW) algorithm is a method proposed to measure the similarity between two temporal sequences and to align them in a more consistent way [43]. Preprocessing of data points with DTW is known to estimate the GMM parameters more precisely so that a more concrete GMR trajectory can be reproduced. Hence, DTW is widely applied in fields where temporal sequences are used, such as video, audio, and graphics data.

A basic idea of DTW is summarized as follows [16]. Given two trajectories ( ξ and $\bar{\xi }$ ) of length *T*, consider the distance between two data points of temporal index $k_{1}$ and $k_{2}$ , i.e., $h\left(k_{1},k_{2}\right)=∥\xi_{k_{1}}-{\bar{\xi }}_{k_{2}}∥$ . Then, DTW determines the warping path ( $S={\left\{s_{l}\right\}}_{l=1}^{L}$ ) for *L* elements of $s_{l}=\left\{k_{1},k_{2}\right\}$ such that its cumulative distance ( $\gamma (k_{1},k_{2})$ ) is successively minimized by the induction process:

$$\begin{array}{cc} \gamma & \left(k_{1},k_{2}\right)=h\left(k_{1},k_{2}\right)+\min\left[\gamma \left(k_{1}-1,k_{2}-1\right),\gamma \left(k_{1}-1,k_{2}\right),\gamma \left(k_{1},k_{2}-1\right)\right], \end{array}$$

with an initial value of γ(1,1)=0 . As discussed later, time alignment through the DTW step is particularly important in implementing GMR for nonprehensile manipulation tasks because the contact force is often impactive (i.e., a large force is applied within a short duration of time).

The actual implementation of background theories presented in this section can be better understood with more specific details in subsequent sections.

## 4. Methods and Approach

In this section, we explain how we implement LfD for multiple modalities of sensory data and how to integrate them into control design for nonprehensile manipulation.

### 4.1. System Overview

**Figure 1:**
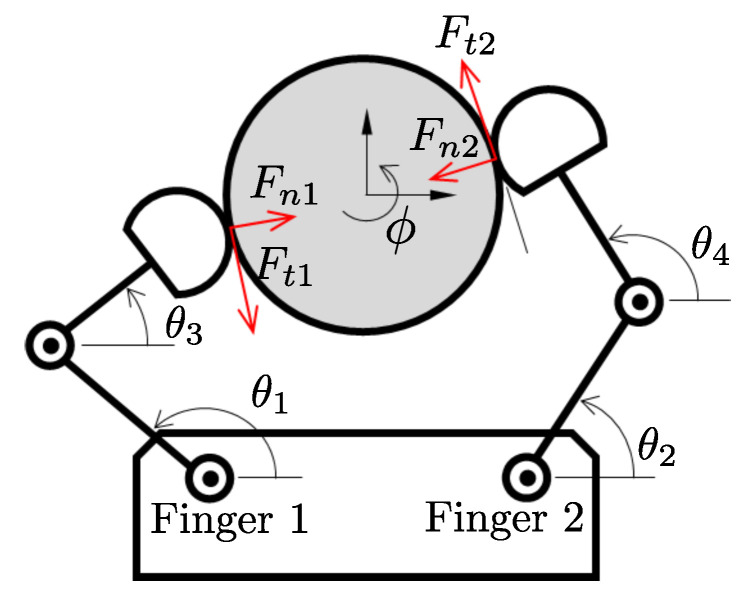
Schematic of the two-finger robot with a circular object.

Figure 1 shows a schematic drawing of the system of concern. A two-finger robotic hand manipulates the circular object. We can measure both the position angles at each joint and the contact force for each finger tip. $F_{n•}$ and $F_{t•}$ denote the normal and tangential components of the contact force, respectively. Note that the tangential components of the two contact forces are expected to be equal in magnitude and opposite in direction, so we consider them separately so that we can monitor and control each individually. The center of the object is fixed so that its motion is constrained to rotate about its axis. ϕ denotes the rotational angle of the circular object. The goal is to rotate the object at the desired angle ( $\phi_{d}$ ). The range of motion of the object is limited to relatively small angles (e.g., $∼45^{\circ }$ ) if we manipulate it with two fingers by maintaining contact with the object at all times (i.e., stable grasping with force closure). To achieve a large-angle rotation, multiple steps of stable regrasping (i.e., finger gaiting) are required, making the manipulation very slow. On the contrary, if we allow the fingers to spin the object by flinging and catching it (i.e., nonprehensile manipulation), a large amount of rotation can be achieved in a much shorter time.

### 4.2. Multisensory Learning from Demonstration

In order to achieve the desired nonprehensile manipulation described above, we first generate multisensory motion profiles with GMM/GMR for both position and contact force. The process consists of four stages: data collection, preprocessing, probabilistic modeling, and generalized trajectory reproduction.

#### 4.2.1. Data Collection

There are various ways for experts to provide demonstrations to the robot. In this work, we focus on kinesthetic teaching, in which a human teacher guides the robot in performing the skill by physically holding it [44]. Kinesthetic teaching was chosen owing to its advantage over other demonstration techniques (e.g., teleoperation and vision systems), such as direct compensation of the kinematic constraints and avoidance of correspondence problems resulting from the direct relationship between the demonstration and readings from sensors on the robot.

**Figure 2:**
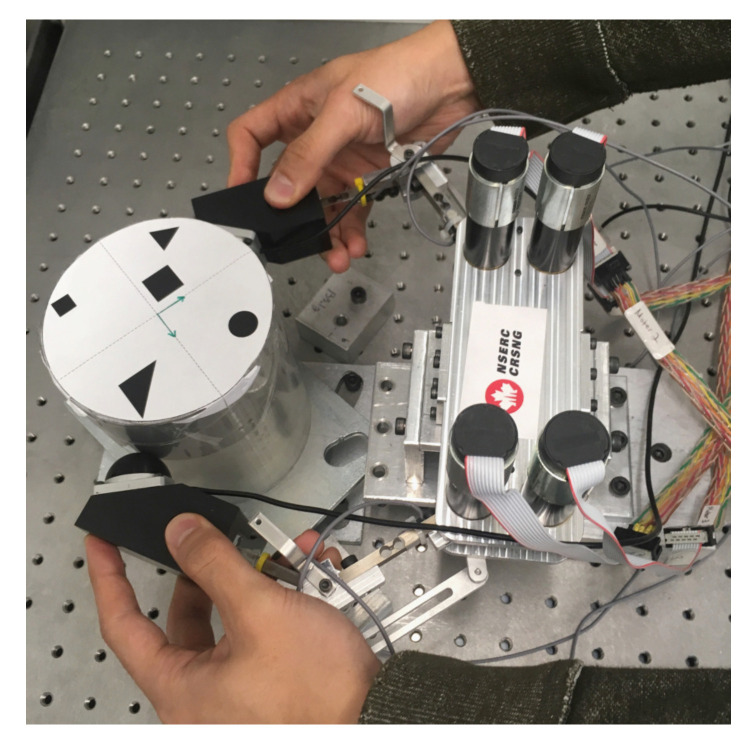
Performing kinesthetic teaching.

Figure 2 shows how the kinesthetic teaching was performed by a human expert. As mentioned above, the LfD framework is employed to allow the robot to learn the joint angles and the end-effector force motion profiles for the impulsive spinning motion. Hence, the demonstration was performed with a particular emphasis on creating a fast spinning action through impulsive force. The range of motion and the amount of force required to generate such a motion exceeded those that can be realized by two fingers (i.e., a thumb and an index finger) of a single hand. Hence, instead of using a single hand, we executed the demonstration using two hands, with each hand holding onto each robot finger, as shown in Figure 2. The rounded rubber surface of the fingertip force sensors facilitated the desired action by providing a large amount of surface friction and a cushioning effect during the impulsive spinning maneuver. Therefore, spinning the object indirectly through robotic fingers provided some advantages over direct contact between the human hand and the object in terms of some ergonomic aspects, such as safety, comfort, and ease of use.

#### 4.2.2. Data Preprocessing for Temporal Alignment

From human demonstrations, we can collect continuous sequences of points (i.e., trajectories) in the state space. For the nonprehensile manipulation task, we are interested in both motion and force trajectories. For each time step (*j*), the dataset is defined by $\xi_{j}^{m}=\left\{{\left(\xi_{t}\right)}_{j}^{m},{\left(\xi_{\theta }\right)}_{j}^{m},{\left(\xi_{f}\right)}_{j}^{m}\right\}$ , where m=1,2,…,M is the index for the number of demonstrations, ${\left(\xi_{t}\right)}_{j}^{m}$ represents the time-step data, ${\left(\xi_{\theta }\right)}_{j}^{m}\in R^{4}$ is the joint angle vector, and ${\left(\xi_{f}\right)}_{j}^{m}\in R^{4}$ is the end-effector (i.e., finger tip) force vector, all for the *m*-th demonstration at time step *j*. Note that we directly used the joint angles instead of Cartesian coordinates of fingertips for motion trajectories because the robotic fingers are controlled by motors with positional PD control at each joint.

**Figure 3:**
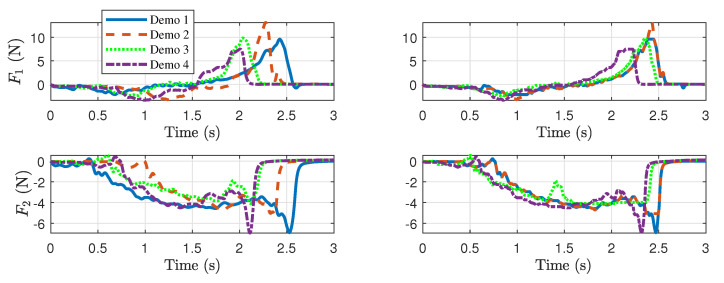
Comparisons of contact forces from kinesthetic teaching before and after DTW. The plots in the left column are raw angles (before DTW), and those in the right column are time-aligned signals (after DTW). The first row corresponds to finger 1, and the second row corresponds to finger 2.

**Figure 4:**
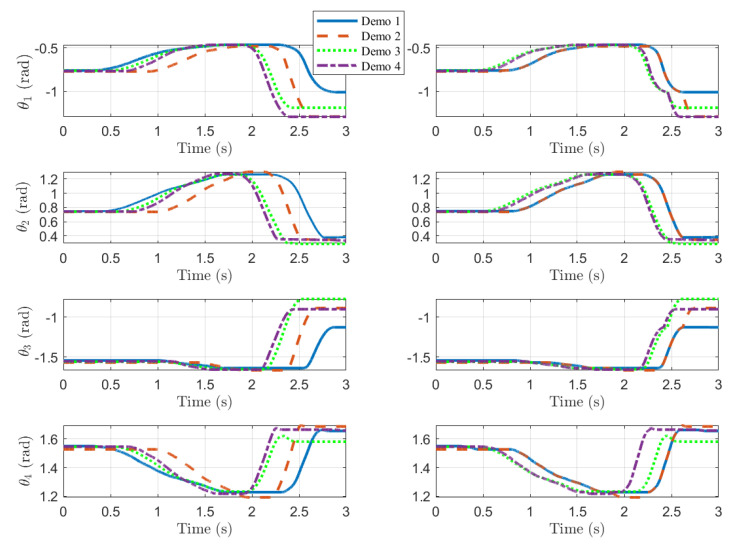
Comparisons of angles from kinesthetic teaching before and after DTW. The plots in the left column are raw angles (before DTW), and those in the right column are time-aligned (after DTW). The denotations of angles are the same as in Figure 1.

The time alignment of both position and force is crucial when performing dynamic dexterous manipulation because the end-effector force must be applied to the object at an appropriate position on the robot fingers to maximize the effectiveness of the dynamic manipulation. Hence, it is very important to align the demonstrated trajectories in time through the DTW algorithm. Since a single DTW algorithm cannot align more than two time series at the same time, we set a single reference trajectory and perform DTW multiple times with the rest of the trajectories. Figure 3 and Figure 4 illustrate the application of DTW in the processing of contact force and position signals, respectively. In each figure, the plots in the left column indicate raw data, and those in the right column represent data after applying DTW.

#### 4.2.3. Trajectory Modeling and Reproduction

The data recorded from the demonstration are then modeled with a GMM. As mentioned in Section 3.1, we select *K* Gaussians to represent the entire trajectory parameterized by $\left(\pi_{k},\mu_{k},\Sigma_{k}\right)$ for k=1,2,…,K .

After the demonstration data are modeled, smooth motion and force trajectories are found using GMR. As explained in Section 3.2, given a joint probability distribution of training data ( $p(\xi_{t},\xi_{\theta },\xi_{f})$ ), the GMR estimates the conditional probabilities for the position ( $p(\xi_{\theta }|\xi_{t})$ ) and the force ( $p(\xi_{f}|\xi_{t})$ ). Then, the expectation of the conditional probability results in smooth motion and force trajectories along the time space.

### 4.3. Motion Control with Multisensory GMR Trajectories

**Figure 5:**
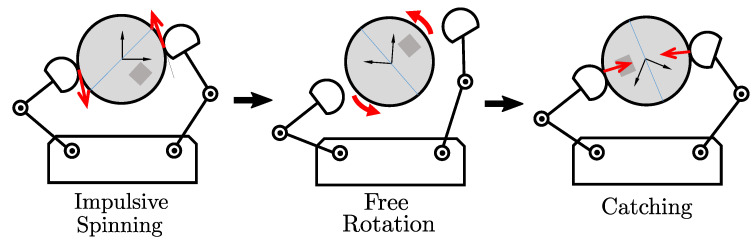
Control procedure for nonprehensile spinning. The arrows indicate direction of forces applied by the finger tips.

Nonprehensile manipulation for spinning proceeds in three steps, as shown in Figure 5: impulsive spinning, free rotation, and catching. The spinning move is the main task required to generate the fast rotational motion in a nonprehensile way. Thus, we employ GMM/GMR for the spinning manipulation.

**Figure 6:**
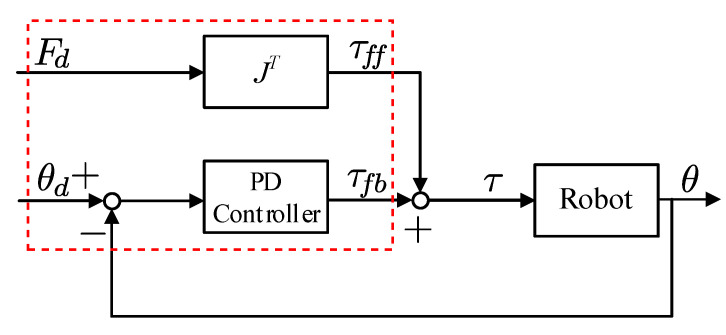
Controller for the spinning move.

For the spinning move, the generalized motion and force trajectories are applied to a robotic hand as control inputs, which are denoted by $\theta_{d}$ and $F_{d}$ , respectively. Since the motion is represented by the joint angles, a simple PD feedback controller is capable of generating the desired finger motion. To perform dynamic dexterous manipulation, additional end-effector forces are often needed. Hence, we perform hybrid control by adding a feedforward term to the PD position controller. To provide the desired end-effector force during the manipulation, we use Jacobian transpose control to calculate the required feedforward portion of the joint torque. Thus, the control input (τ) to the robot hand is expressed as

$$\tau =\tau_{ff}+\tau_{fb}$$

where $\tau_{ff}$ and $\tau_{fb}$ are the feedforward and feedback control terms, respectively, which are expressed as

(9) $$\begin{array}{cc} \tau_{ff} & =J^{⊺}\left(\theta \right)F_{d}\\ \tau_{fb} & =K_{P}\left(\theta_{d}-\theta \right)+K_{D}\left(\dot{\theta_{d}}-\dot{\theta }\right) \end{array}$$

where $J\in R^{4\times 4}$ denotes the Jacobian matrix, and $K_{P}\in R^{4\times 4}$ and $K_{D}\in R^{4\times 4}$ are diagonal matrices that contain the proportional and derivative gains for each joint, respectively. A block diagram of the controller for the spinning move is presented in Figure 6. The control law in Equation (9) is indicated by a dotted red box in Figure 6.

After the robot performs an impulsive spin, the visual feedback is used to stop the spinning object at a desired angle ( $\phi_{d}$ ). The stopping action involves dynamic catching (or an impulsive grasping action) by sending a simple closing command to both fingers. ${\bar{\phi }}_{t}$ denotes the rotation angle of the object measured by the vision sensor at time step *t*, and the time to trigger the regrasping action is denoted by $t_{tr}$ and can be determined by the following equation:

(10) $$t_{tr}=t\;\mathrm{such}\mathrm{that}\;{\bar{\phi }}_{t}=\phi_{d}-\omega T_{v}-T_{a}$$

where ω is the angular velocity of the object, $T_{v}$ is the sampling time of the vision sensor, and $T_{a}$ is the time required for both fingers to complete their closing action. We assume that the object is rotating quasistatically, i.e., at a relatively constant angular speed (which holds true for the experiments described in the next section). However, it may also be computed online through the numerical differentiation of successive vision data on the orientation of the object.

## 5. Experimental Results

In this section, we present experimental results to verify the performance of the proposed control strategy applied to the dynamic spinning task.

### 5.1. Experimental Setup

**Figure 7:**
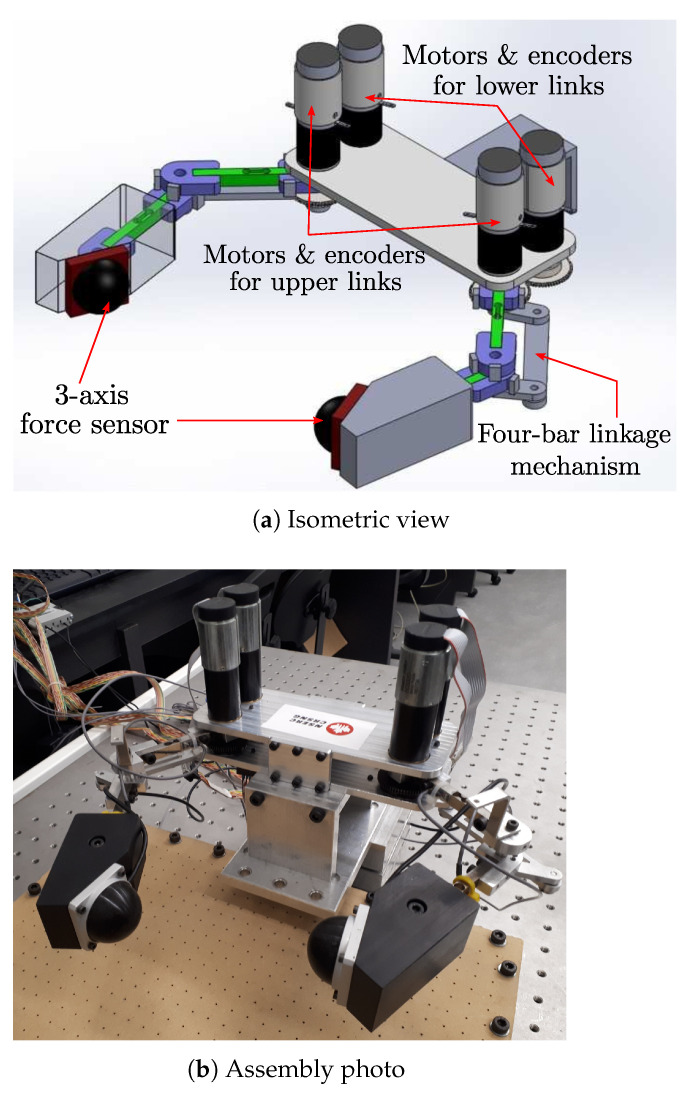
3D schematic and photo of the robotic hand.

**Figure 8:**
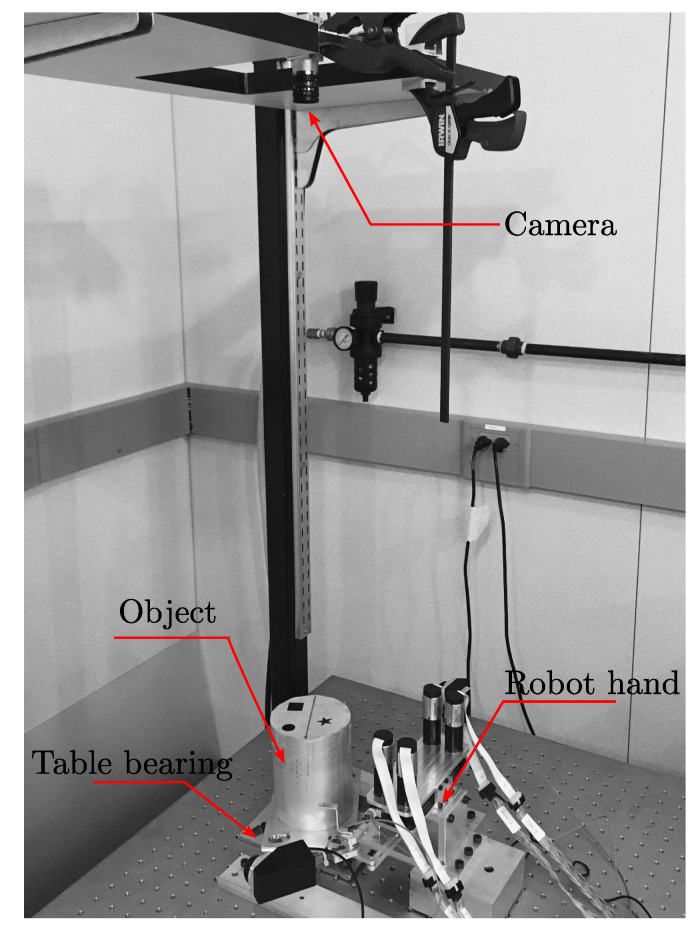
Overall configuration of the experimental setup.

The hardware platform for this research is the custom-built planar robotic hand depicted in Figure 7 and Figure 8. This robot has two fingers, each of which has two degrees of freedom (2 DOFs), consisting of two joints and two links. In order to mimic the sensory-rich behavior of humans, the robot is equipped with multiple sensors; each joint is equipped with an encoder, each arm is a force sensor (strain gauge), and the finger tip has a 3D tactile sensor attached to the end effector. The vision camera at the top of the system (see Figure 8) captures the manipulation scene and measures the configuration of the object in real time. All hardware is connected to LabVIEW Real-Time target (manufactured by National Instruments Inc., Austin, TX, USA) running at 1 kHz, except for the vision loop, which runs at 12.5 Hz ( $T_{v}=80$ ms). The detailed hardware specifications are shown in Table 1.

The position feedback controller is a simple joint PD controller that utilizes the error between the desired and reference angles and their derivatives.The feedback control gains are set as $K_{P}=\mathrm{diag}\left(20,\;20,\;20,\;20\right)$ and $K_{D}=\mathrm{diag}\left(1500,1500,1500,1500\right)$ the unit of which is in analog voltage to the motor drive. The conversion factor from the drive voltage to the motor torque in 0.1233 Nm/V. These gains were selected by trial and error in order to ensure a time response that is fast enough to realize the impulsive spinning task without excessively saturating the motor torque. Note that the PD gains are diagonal matrices because our controller has a decentralized PD control structure for each robotic finger. For more details on the performance evaluation of the servo controller, as well as the feedforward controller, see [45].

As shown in Figure 8, the object is mounted on a table bearing to reduce the friction against the ground as much as possible. An image identifier tag is attached on top of the object to allow the vision camera to track the orientation of the object. The cylindrical object is made of aluminum, with a rotational inertia of around 0.057 kg·m^2^. Using our experimental setup, the maximum rotation achieved by a single-step force-closure manipulation is ∼ $\pm 25^{\circ }$ .

### 5.2. Experimental Results

Experiments were conducted to realize fast rotation of the object beyond the range achievable by the single-step stable grasping manipulation. Specifically, we chose three different desired rotation angles: $\phi_{d}=90^{\circ },120^{\circ }$ , and $180^{\circ }$ . Note that the same impulsive spinning move can be used for any $\phi_{d}$ because the particular values of $\phi_{d}$ can be achieved by varying the values of $t_{tr}$ in Equation (10) (i.e., the time to engage in the stopping action). Thus, we obtained only one set of generalized trajectories of position and force for the spinning move.

To find generalized trajectories, we collected a total of four demonstration data points (i.e., M=4 ) by holding the robot with human hands and performing kinesthetic teaching, during which the sensor readings were recorded. As we controlled the robot’s position using position tracking, we sent the encoder readings directly to our position trajectory. The original force readings are converted to normal and tangential components at the contact point. In learning the force profile, we only used the tangential force. There are several reasons for this: the task is limited to only rotation; hence, the normal direction force does not play much of a role in spinning. Furthermore, since the object center is fixed, the normal force does not reflect the actual object force.

**Figure 9:**
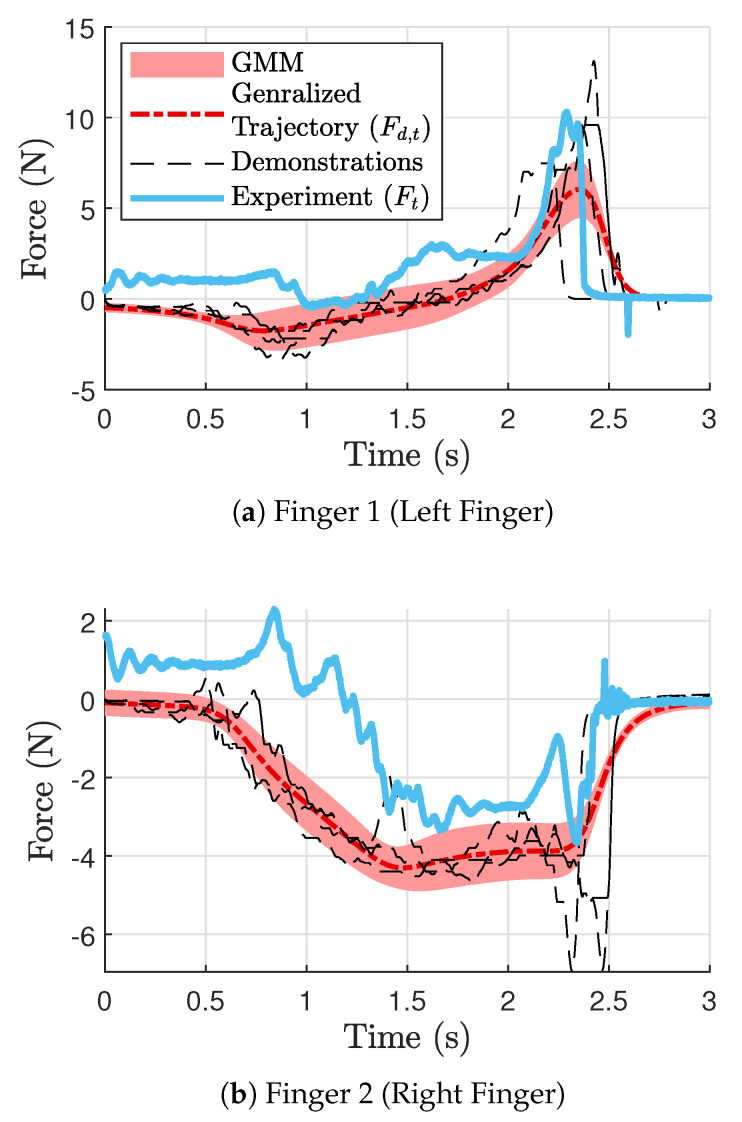
GMR trajectories for tangential forces [45].

**Figure 10:**
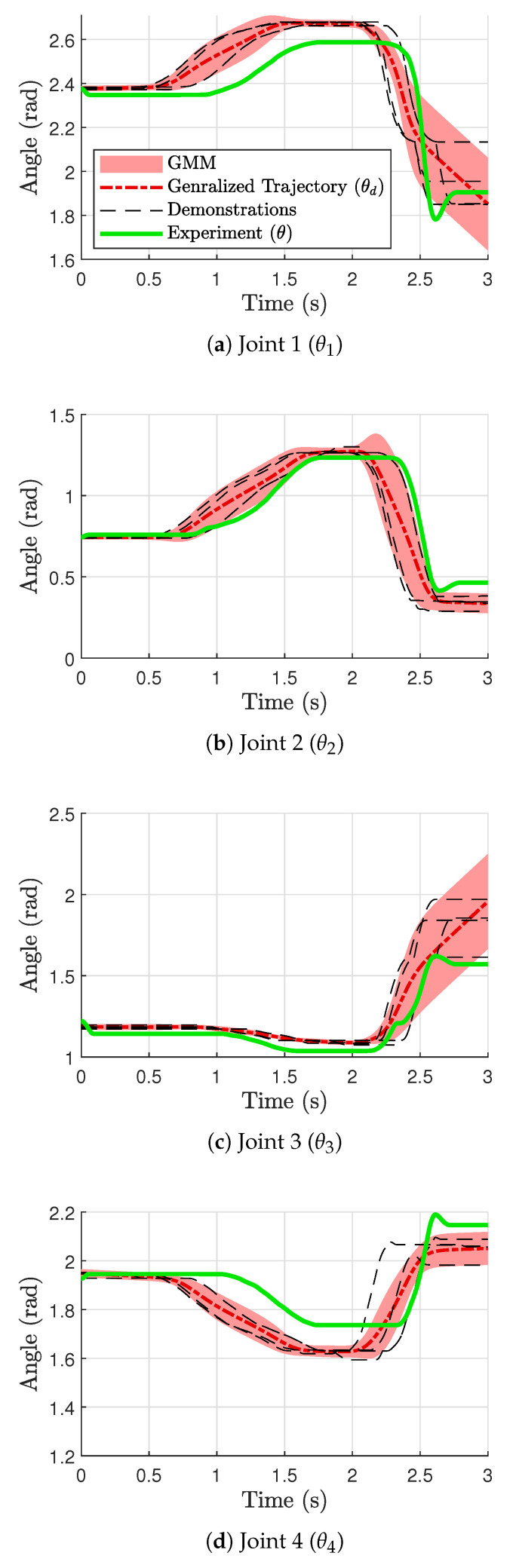
GMR trajectories for position variables.

The resulting generalized trajectories are shown in Figure 9 (force) and Figure 10 (position). Figure 9a and Figure 10a,c correspond to finger 1, while Figure 9b and Figure 10b,d correspond to finger 2 (See Figure 1). The thin dotted lines represent the demonstration data from kinesthetic teaching (four in each plot). The thick dashed–dotted line is the generalized trajectory obtained through GMM, and the gray area around them represents the corresponding variances. We used five component Gaussian distributions (i.e., K=5 ) for the GMMs. The generalized trajectories shown in Figure 9 and Figure 10 were implemented in the controller shown in Figure 6, and the resulting signals are depicted as thick solid lines in Figure 9 and Figure 10. Note that the fingers are in contact with the object from around 0.8 s until around 2.7 s.

**Figure 11:**
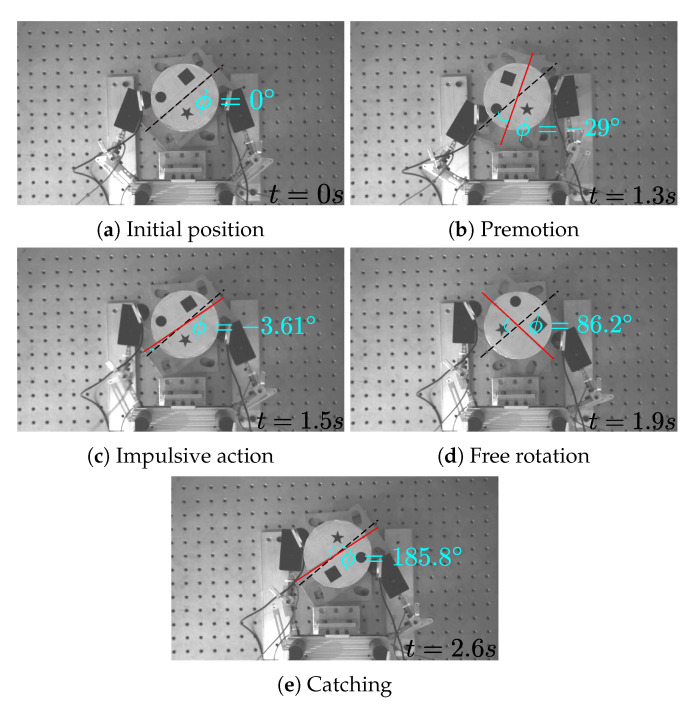
Snapshots of the manipulation for $\phi_{d}=180^{\circ }$ Circle, square and star markers on the object are used for image processing [45].

**Figure 12:**
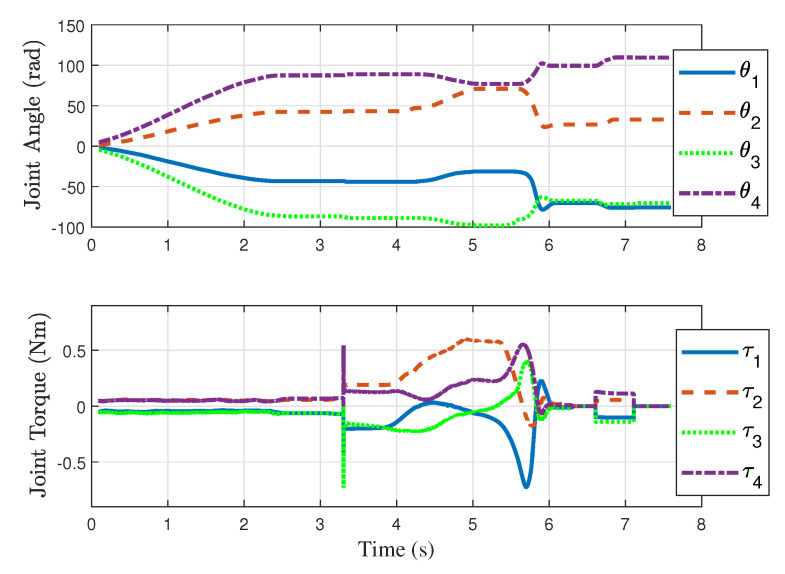
Joint angles and joint torques corresponding to the experiment that generated snapshots shown in Figure 11.

To illustrate the whole procedure of the nonprehensile spinning manipulation, snapshots for $\phi_{d}=180^{\circ }$ are shown in Figure 11, which were captured by the vision camera. The five snapshots in Figure 11 delineate different stages of motion that constitute the complete nonprehensile spinning manipulation. As shown in Figure 11a, first, both fingers are moved to the object so that they grasp it, symmetrically at the rightmost and the leftmost contact points located along the horizontal line passing through the object center. Then, both fingers are engaged in the wind-up motion (called the premotion), as shown in Figure 11b, whereby the object is slowly rotated counterclockwise through a finger tip rolling to its maximum range ( $∼-29^{\circ }$ in this case) to be ready for the impulsive spinning move. Then, both fingers quickly fling the object in the opposite (i.e., clockwise) direction through impulsive spinning, as shown in Figure 11c, which depicts a snapshot of the moment when both fingers have just spun the object with a quick motion. Then, a period of free motion follows, as shown in Figure 11d. During this period, the object undergoes constant-speed rotation while the fingers remain detached from the object. Finally, as shown in Figure 11e, when the object approaches its desired rotation ( $\phi_{d}$ , which is $180^{\circ }$ in this case), the fingers are engaged in the fast catching action to stop the rotation according to Equation (10). The joint torques and the joint angles corresponding to Figure 11 are also shown in Figure 12.

We conducted 10 repeated trials for each $\phi_{d}$ to evaluate the performance of the robotic hand. The performance of the nonprehensile spinning manipulation is compared with that of finger gaiting in Table 2 and Table 3. For all three values of $\phi_{d}$ , the nonprehensile manipulation completed the rotation task within 4 s, as shown in Table 2. On the hand, it takes a significantly longer time if we use the classical finger gaiting. Specifically, it takes about 20 s to rotate the object 90 degrees, as shown in Table 3. This is because the robotic fingers need to complete four rounds of regrasping before completing the desired rotation. Of course, the time of completion increases in proportion to $\phi_{d}$ , e.g., 35 s are required to complete $180^{\circ }$ of rotation. Although the angle errors appear to be smaller for finger gaiting, the error values reported in Table 2 are insignificant because we can easily compensate for all these errors by incorporating a small rolling manipulation followed by the catching motion (which takes only a fraction of a second). The recorded videos of the nonprehensile manipulation, as well as conventional finger, gating can be viewed in [46,47], respectively.

## 6. Concluding Remarks

In this paper, we proposed the use of the multisensory learning from demonstration (LfD) framework to enable nonprehensile (or dynamic dexterous) manipulation tasks with a multifingered robotic hand. The main idea is to produce generalized trajectories for both position and force using a GMM/GMR-based LfD technique. Then, the force data from the GMR are implemented as a feedforward command, while the position data are used for feedback control. We demonstrated the performance of the proposed manipulation technique through experimental tests with a two-finger planar robotic hand, which was controlled to spin a circular object rapidly and accurately. The proposed technique was also compared with the classical regrasping method based on force closure and finger gaiting, which showed the superiority of our approach in terms of speed and agility. We believe that the proposed framework can be generalized to other nonprehensile manipulation tasks that involve more complex dynamics between the object and the manipulating bodies.

We employed a simple (decentralized) PD control law for the position tracking reported in this paper. Using more advanced control techniques (e.g., nonlinear control methods) may further improve the performance of the manipulation. As demonstrated by the experimental results, the proposed control strategy can substantially shorten the processing time required to manipulate a target object with superior repeatability. Hence, the proposed strategy has considerable potential for industrial vision-based manipulation tasks where we need to process a large number of similar objects at a fast pace.

## Figures and Tables

**Table 1:** Hardware specifications [45].

Component	Manufacturer/Model	Specification
Geared Motor Set	Maxon Motor (Sachseln, Switzerland) (222053, 201937, 201937)	Max speed: 9270 rpm Rated torque: 11.6 mNm Gear ratio: 84:1 Encoder resolution: 512 ppr
Strain Gauge	Strain Measurement Device (Wallingford, CT, USA) (S220)	Max load: 6 lbs
Three-Axis Force Sensor	OnRobot (Budapest, Hungary) (OMD-30-SE-100N)	Nominal capacity: 100 N ( $F_{z}$ compression), ±25 N ( $F_{xy}$ )
Vision Sensor	Basler (cA2000-340km)	Resolution: 2048 px × 1088 px

**Table 2:** Performance of nonprehensile manipulation to generate the desired angle ( $\phi_{d}$ ).

Desired Angle	Average Final Angle	Average Time of Completion	Std. Dev. of Angle Error
90°	91.63°	3.46 s	2.007°
120°	119.4°	3.97 s	2.1739°
180°	182.1°	3.77 s	2.4612°

**Table 3:** Performance of finger gaiting to generate the desired angle ( $\phi_{d}$ ).

Desired Angle	Average Final Angle	Average Time of Completion	Std. Dev. of Angle Error
90°	91.03°	19.520 s	0.464°
120°	120.8°	22.109 s	0.355°
180°	180.4°	35.782 s	0.450°

## Data Availability

No new data were created or analyzed in this study. Data sharing is not applicable to this article.
